# Imaging features of primary Sarcomas of the great vessels in CT, MRI and PET/CT: a single-center experience

**DOI:** 10.1186/1471-2342-13-25

**Published:** 2013-08-07

**Authors:** Christian von Falck, Bernhard Meyer, Christine Fegbeutel, Florian Länger, Frank Bengel, Frank Wacker, Thomas Rodt

**Affiliations:** 1 Department of Radiology, Hannover Medical School, Hannover 30625, Germany; 2 Department of Cardiothoracic, Transplant and Vascular Surgery, Hannover Medical School, Hannover, Germany; 3 Institute of Pathology, Hannover Medical School, Hannover, Germany; 4 Department of Nuclear Medicine, Hannover Medical School, Hannover, Germany

## Abstract

**Background:**

To investigate the imaging features of primary sarcomas of the great vessels in CT, MRI and ^18^ F-FDG PET/CT.

**Methods:**

Thirteen patients with a primary sarcoma of the great vessels were retrospectively evaluated. All available images studies including F-18 FDG PET(/CT) (n = 4), MDCT (n = 12) and MRI (n = 6) were evaluated and indicative image features of this rare tumor entity were identified.

**Results:**

The median interval between the first imaging study and the final diagnosis was 11 weeks (0–12 weeks). The most frequently observed imaging findings suggestive of malignant disease in patients with sarcomas of the pulmonary arteries were a large filling defect with vascular distension, unilaterality and a lack of improvement despite effective anticoagulation. In patients with aortic sarcomas we most frequently observed a pedunculated appearance and an atypical location of the filling defect. The F-18 FDG PET(/CT) examinations demonstrated an unequivocal hypermetabolism of the lesion in all cases (4/4). MRI proved lesion vascularization in 5/6 cases.

**Conclusion:**

Intravascular unilateral or atypically located filling defects of the great vessels with vascular distension, a pedunculated shape and lack of improvement despite effective anticoagulation are suspicious for primary sarcoma on MDCT or MRI. MR perfusion techniques can add information on the nature of the lesion but the findings may be subtle and equivocal. F-18 FDG PET/CT may have a potential role in these patients and may be considered as part of the imaging workup.

## Background

Primary malignant sarcomas of the great vessels are exceedingly rare, representing less than 1% of all sarcomas [1,2]. Hence, information on these malignancies is restricted to case reports and small retrospective series. The most frequent sites of origin are the pulmonary arteries and the aorta, followed by venous sarcomas, predominantly of the inferior vena cava [1]. Pulmonary artery sarcomas commonly arise from the pulmonary trunk and the central left or right pulmonary artery. The most dominant histologic subtypes of sarcomas of the great vessels as reported in the literature are undifferentiated intimal sarcoma and leiomyosarcoma. The subtyping of sarcomas has changed in the last decades primarily by the evolvement of modern immunohistological techniques thus making comparisons between published historical and contemporary studies difficult. In both, pulmonary and aortic location, an intraluminal growth pattern is differentiated from a less frequent mural growth type [1-3].

The prognosis of patients with primary malignant sarcomas of the great vessels is poor, mainly due to the late presentation of these patients with locally advanced disease or distant metastases. Clinical features may resemble those of pulmonary or aortic thromboembolic disease [1-3]. The diagnosis may be further delayed by a misinterpretation of imaging studies of this rare disease, e.g. pulmonary artery sarcoma is interpreted as pulmonary embolism [3-5].

The aim of this study was to retrospectively identify typical imaging features suggestive of primary malignancy of intrathoracic vessels in combined F-18-fluorodesoxyglucose positron emission tomography/computed tomography (F-18 FDG PET/CT), multidetector computed tomography (MDCT) and magnetic resonance imaging (MRI). We therefore analyzed the imaging appearance of primary sarcomas of the great vessels in F-18 FDG PET/CT, MDCT and MRI as seen in 13 consecutive patients who were treated in a single tertiary care university center.

## Methods

### Study population

This retrospective study was conducted in accordance with the guidelines of the Declaration of Helsinki and according to standards of the local ethics committees. The ethical committee of Hannover Medical School waived the need for written informed consent because routine diagnostic data was analyzed anonymously.

Between 2002 and 2011 a total of 13 patients (m = 7, f = 6) with histologically proven primary sarcoma of the great vessels were treated in a single tertiary care university hospital. The mean patient age was 57.5 (±12.2) years, ranging from 46 to 81 years. All available imaging studies of these patients including F-18 FDG PET/CT (*n* = 3), F-18 FDG PET (*n* = 1), MDCT (*n* = 12) and MRI (*n* = 6) were evaluated by two experienced readers in consensus and typical imaging features of this rare tumor entity were identified.

### Imaging studies

The PET/CT scans were acquired 90 minutes after the intravenous application of 5 MBq/Kg body weight of F-18-2-fluoro-2-desoxyglucose (F-18 FDG) on a dedicated dual-slice hybrid imaging system (Siemens biograph 2, Forchheim, Germany) in three patients and on a PET-only scanner in one case (Siemens ECAT Exact, Knoxville, TN, USA). The acquisition time was five minutes per bed position. A total of 7 – 8 bed positions were scanned to cover a region from the vertex to the upper thigh. A co-registered low-dose CT was used for attenuation correction and anatomical localization (tube voltage = 130 kVp, tube current = 20mAs_eff_ (modulated), slice collimation = 2 x 5 mm, pitch = 1.5, reconstruction increment = 2.5 mm, reconstruction kernel = B30s). Neither oral nor intravenous contrast agents were administered for the combined PET/CT scan.

All CT scans were acquired on MDCT scanners with 4 – 64 simultaneously acquired sections (Siemens Somatom Volume Zoom/Sensation 16/Emotion 16/Sensation 64, Forchheim, Germany; General Electric VCT, Chalfont St. Giles, UK; Toshiba Aquillion, Otawara, Japan). The section thickness was in the range from 0.625 mm to 5 mm and the reconstruction increment in the range from 0.5 mm to 4 mm. The tube voltage was 120 kVp for all examinations. Dose modulation was used with all scanners. The MDCT scans were performed after the power injection of 80-100 ml of an anionic iodinated contrast agent at a flow of 3-4 ml/s followed by a saline chaser. The scan timing was adjusted to maximize the contrast in the region of interest, i.e. the pulmonary arteries or the thoracic and abdominal aorta. The images were reconstructed with standard abdomen kernels, supplemented by an additional high-resolution reconstruction kernel for chest scans. Multiplanar reformations (MPR) in the coronal and sagittal orientation were available in all cases.

The MRI scans were acquired on scanners from three different manufacturers with a field strength of 1.5 T (Siemens Avanto, Erlangen, Germany; Philips Intera, Best, The Netherlands; General Electric CV/i, Chalfont St. Giles, UK). All examinations included T1- and T2-weighted sequences before and T1-weighted sequences with spectral fat suppression after the injection of a gadolinium-based contrast agent as well as a dynamic contrast-enhanced ultra-fast gradient echo acquisition or an MR-angiography (MRA) sequence. Three examinations were supplemented with an additional inversion-recovery (IR) sequence for the detection of late enhancement (LE) and three scans included ECG-gated cine-sequences (FIESTA/Cine Trufi).

### Histopathologic diagnosis

The surgically derived specimens were embedded into paraffin and sectioned according to standard histopathologic procedures. Routine staining included hematoxylin and eosin (H&E), Elastica van Gieson (EvG) and periodic acid-schiff (PAS). The diagnosis was supplement by additional immunohistochemical stains. The tumors were classified according to the current WHO classification of soft tissue tumors [6].

## Results

### Patient demographics and histopathologic diagnosis

A total of 13 patients were included in this retrospective study (m = 7, f = 6). The mean age was 57 (±12) years. With respect to primary *pre-operative* imaging studies MDCT scans were available in 12, MR scans in 6, F-18 FDG PET/CT scans in 3 cases and an F-18 FDG PET scan in one case. The primary tumors were located at the right (*n* = 5), left (*n* = 1) or central (*n* = 3) pulmonary artery (*n* = 9 in total), the right inferior pulmonary vein (*n* = 1), the aortic arch (*n* = 1), the descending aorta (*n* = 1) and the abdominal aorta (*n* = 1). Tumor histology was undifferentiated intimal sarcoma (*n* = 8), angiosarcoma (*n* = 2), leimyosarcoma (*n* = 2) and myxofibrosarcoma (*n* = 1). This information is summarized in Table 1.

**Table 1:** Patient demographics and diagnosis

**Patient #**	**Age**	**Sex**	**Location**	**Histology**	**Primary diagnosis**
1	74	M	Left pulmonary artery	UIS	Pulmonary Embolism
2	45	M	Central pulmonary artery	UIS	Pulmonary Embolism
3	68	M	Descending aorta	UIS	Atheromatous Disease/Chronic Dissection
4	81	M	Abdominal aorta	AS	Contained Aortic Rupture
5	51	F	Right pulmonary artery	UIS	Pulmonary Embolism
6	46	M	Pulmonary vein	LMS	Pneumonia
7	71	M	Distal aortic arch	AS	Congestive Heart Failure/Thrombus
8	49	F	Right pulmonary artery	MFS	Pulmonary Embolism
9	55	F	Right pulmonary artery	UIS	Pulmonary Embolism
10	46	F	Central pulmonary artery	UIS	Pulmonary Hypertension
11	47	F	Central pulmonary artery	UIS	Pulmonary Embolism
12	61	F	Right pulmonary artery	LMS	Pulmonary Embolism
13	54	M	Right pulmonary artery	UIS	Pulmonary Embolism

We observed a median interval between the first imaging study and the final diagnosis of 11 weeks, ranging from 0 to 12 weeks. However, the interval between the first contact to a physician due to clinical symptoms that could retrospectively attributed to the final diagnosis was much longer in three cases with 30 weeks (dyspnea), 52 weeks (congestive heart failure) and 30 weeks (dyspnea), respectively. The primary tentative diagnoses were pulmonary embolic disease (*n* = 8) and idiopathic pulmonary hypertension (*n* = 1) in the patients with a tumor in the pulmonary arteries. In cases with the tumor being located in the aorta, the tentative diagnoses were atheromatous disease and chronic dissection in two cases. The patient with the sarcoma of the right inferior pulmonary vein was primarily treated for pneumonia as the venous infarction as seen on a chest x-ray was interpreted as a consolidation. One patient underwent an MDCT scan because of B-symptoms including fever, malaise and unintended weight-loss. The MDCT-findings were suggestive of a contained rupture of an inflamed aorto-bi-iliac prosthesis and the patient underwent immediate surgical treatment.

### Imaging characteristic

All available F-18 FDG PET(/CT), MDCT-, MRI- and studies were reviewed with respect to the presence of imaging features suggestive of primary malignancy of the vascular filling defects. In patients with a sarcoma of the pulmonary artery (*n* = 8), we observed the following suspicious imaging features in varying frequency: a filling defect of the entire vessel diameter with vascular distension (*n* = 8/8), a large *unilateral* ‘thrombus’ (*n* = 7/8), lack of clinical improvement despite adequate antico-agulation without evidence of deep venous thrombosis (*n* = 4/8), an expansion beyond the vessel wall (*n* = 2/8, in the follow-up examination), a heterogeneous enhancement after the intravenous administration of a contrast agent in MRI (*n* = 3/4, MRI was available in 4 patients), metabolic activity as demonstrated by F-18 FDG PET(/CT) (*n* = 3/3, PET was available in 3 patients), a pedunculated appearance (*n* = 1/8), and local or distant metastases (*n* = 2/8).

The semiquantitative analysis of the metabolic activity of the tumors in F-18 FDG PET/CT revealed SUV_max_-values of 16.1, 8.8 and 14.5, respectively. There was no additional information gathered from the late enhancement sequences. In the patients with sarcomas of the aorta (*n* = 3) we observed comparable imaging features, however, in a different frequency: a pedunculated appearance (*n* = 2/3), an atypical location for a thrombus (n = 2), an expansion beyond the vessel wall (*n* = 1/3), contrast enhancement in MRI (*n* = 2/2, MRI was available in 2 patients) or MDCT (n = 1/1, MDCT was available in 1 patient) and metabolic activity as demonstrated by F-18 FDG PET/CT (*n* = 1/1; SUV_max_ = 3.6 – 5.5, PET was available in 1 patient). In the patient with pulmonary vein sarcoma we observed a large filling defect with vascular distension, comparable to the findings as seen in pulmonary artery sarcoma. The related lung parenchyma shows excessive consolidation, consistent with venous infarction.

**Figure 1:**
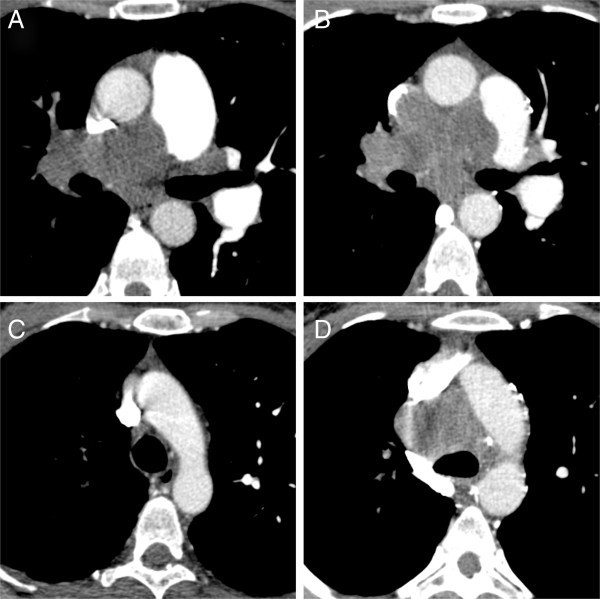
**CT pulmonary angiography (CTPA) of a 49-year-old female patient with a myxofibrosarcoma of the right pulmonary artery.** Suspicious imaging features were already present in the primary imaging **(A**, **C)**. However, the patient was treated conservatively for suspected pulmonary embolism. A follow-up exam was acquired three month later and demonstrated massive disease progression with tumor expansion beyond the vessel wall and local lymph node metastases **(B**, **D)**

As deducible from the relative frequency of the above-mentioned findings, we found a *combination* of indicative imaging features in most patients. In the CTA examinations of the pulmonary arteries in three patients, we observed large, unilateral right-sided filling defects in the pulmonary arteries that obturate the whole cross-sectional area of the vessel and lead to a vascular expansion (Figure 1). However, pulmonary embolism was the primary tentative diagnosis in all three cases and the patients received accordant conservative treatment. The follow-up examinations showed a progression of the findings (Figure 1) or at least a lack of improvement, respectively, despite a sufficient anticoagulation therapy and hence strongly suggest a neoplastic nature of the filling defects.

**Figure 2:**
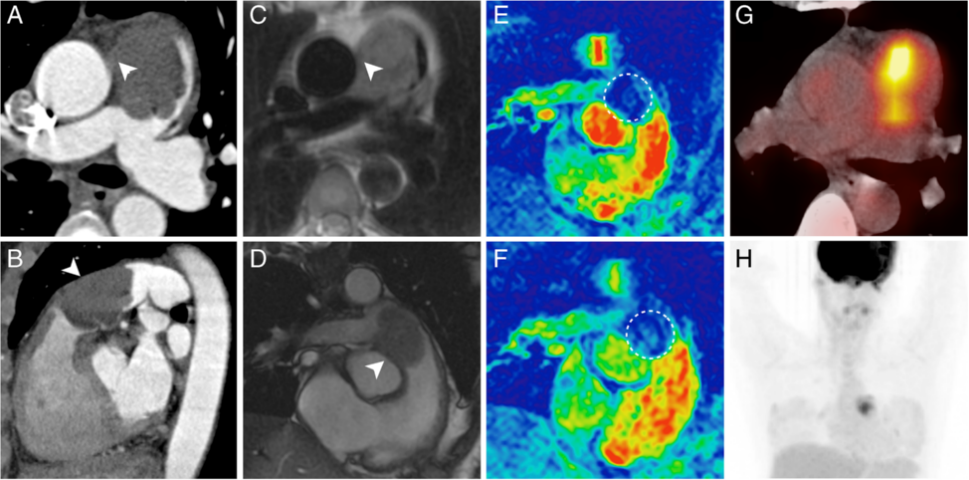
**This figure illustrates an in-depth preoperative work-up in a 46-year-old female patient with an undifferentiated intimal sarcoma of the central pulmonary artery.** Both, the MDCT **(A**, **B)** and the MRI **(C**, **D)** clearly demonstrate a subtotal occlusion of the pulmonary trunk due to an intraluminal process. Imaging findings are suggestive of a focal expansion beyond the vessel wall (A-D, arrowheads). The MR first-pass perfusion sequence **(F)** demonstrates a subtle perfusion of the lesion as compared to the non-enhanced control scan **(E**, circle**)**. The F-18 FDG PET/CT proves a high metabolic activity (SUV_max_ = 7.8) within the lesion **(G**, **H)** and strengthens the suspicion of malignant disease.

The demonstration of lesion vascularization using first pass perfusion or dynamic contrast-enhanced MRI tech-niques can be challenging. We observed only subtle and inhomogeneous contrast enhancements in our patient cohort. Color-coding of the perfusion images may facilitate the perception of the subtle signal changes (Figure 2).

**Figure 3:**
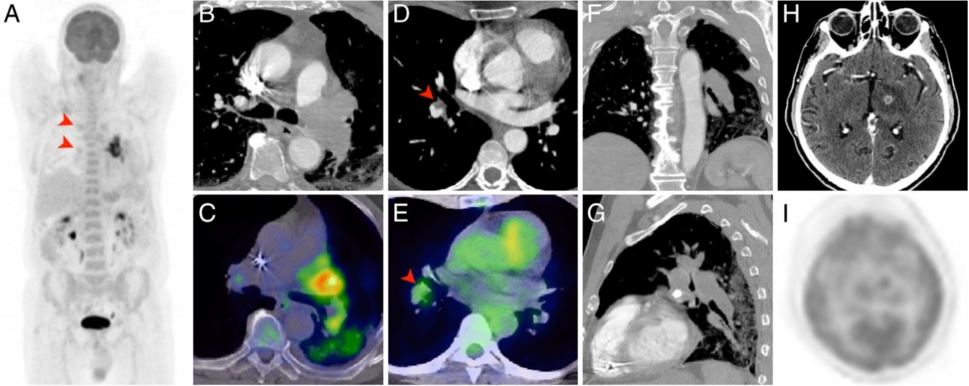
**This figure illustrates the imaging findings in a 74-year-old male patient with an extensive undifferentiated intimal sarcoma of the left pulmonary artery (A-C**, **F**, **G).** Both, the MDCT and the F-18 FDG PET/CT readily demonstrate the local embolic spread in the pulmonary vasculature of the right lung **(A**, **D**, **E**; arrowheads**)** as well as a distant metastasis to the brain **(H**, **I)**.

The evidence of hypermetabolism of the filling defect as shown by F-18 FDG PET/CT, however, could be readily and unequivocally appreciated in all our patients with metabolic imaging (Figures 2, 3) and strengthened the suspicion of malignant disease. Due to the decisive implications of a radical surgical approach, a combination of different imaging studies was requested in many patients of our cohort. The complementary information of morphological, functional and metabolic imaging increases the diagnostic confidence and may facilitate the therapeutic decisions as shown in Figure 3. In equivocal cases, a whole-body staging such as PET/CT may further increase the probability of a malignant disease by revealing distant metastases (Figure 3).

**Figure 4:**
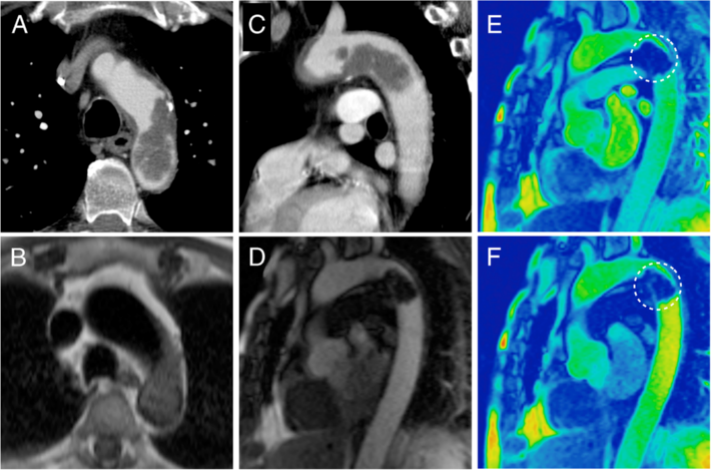
**This figure shows an example of an aortic angiosarcoma of the distal aortic arch in a 71-year-old male patient who was treated for congestive heart failure.** The MDCT and MRI images **(A**-**D)** nicely illustrate the typical pedunculated appearance of the lesion in a location that is atypical for atheromatous thrombi. The first pass perfusion sequence **(F)** suggests vascularisation of the lesion as compared to the unenhanced control acquisition **(E**, circle**)**.

**Figure 5:**
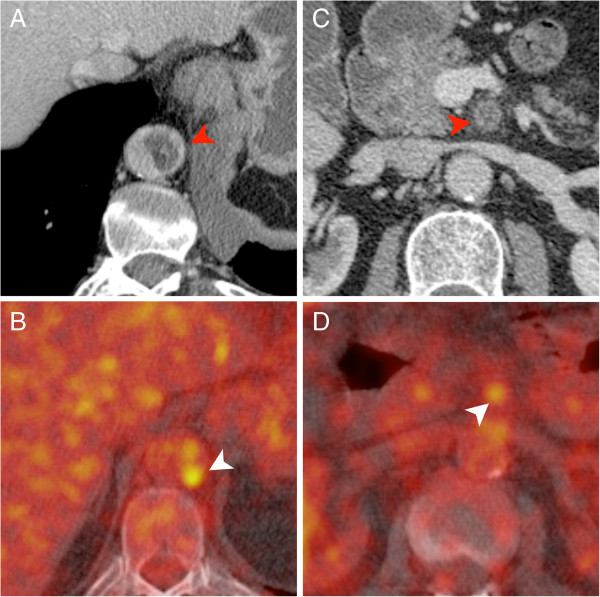
**MDCT and PET/CT of a 71-year-old male patient with an undifferentiated intimal sarcoma of the descending aorta and extension into the superior mesenteric artery (A**, **C).** The PET/CT adds valuable information about the metabolic status of vascular filling defect and strengthens the suspicion of malignant disease **(B**, **D)**.

**Figure 6:**
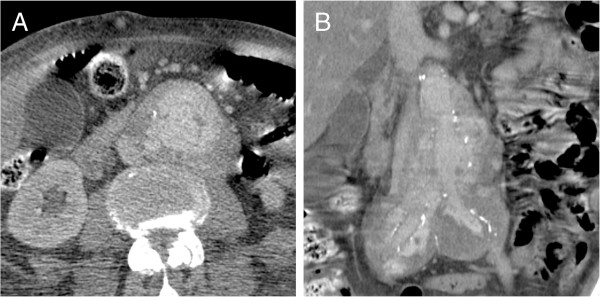
**MDCT images in axial (A) and coronal (B) orientation of a 81-year-old patient with an angiosarcoma of the abdominal aorta who had previously undergone open aortic surgery.** The extensive extramural tumor formation is clearly visualized and may be confounded with chronic inflammatory disease and contained rupture.

Although few in number, our cases with sarcomas located in the aorta suggest that the typical imaging features are comparable to those seen in tumors occurring in the pulmonary vasculature. Potentially suspicious imaging findings of intraaortal filling defects include an atypical location for a thrombus, a pedunculated appearance and a subtle enhancement during first-pass perfusion (Figure 4) and hypermetabolism in F-18 FDG PET/CT (Figure 5), consistent with the intraluminal type of aortic sarcoma. Mural-type sarcomas show different imaging characteristics with predominant extraluminal perivascular growth and can be mistaken for inflammatory disease (Figure 6). The findings are summarized in Table 2.

**Table 2:** Imaging features of primary sarcoma of the great vessels

**Basic imaging features**	
Pulmonary artery sarcoma	Aortic sarcoma
Large *unilateral* obstruction of the main pulmonary arteries	Pedunculated or lobulated appearance
	Unusual location
Vascular distension	
	Expansion beyond the vessel wall
Expansion beyond the vessel wall	
Lack of improvement of a ‘pulmonary embolism’ despite adequate anticoagulation	
No evidence of a deep-venous thrombosis	
Imaging findings during detailed work-up	
High metabolic activity as demonstrated by F-18 FDG PET or PET/CT	
Lesion vascularization (as demonstrated by contrast-enhanced dynamic or perfusion MRI or CT)	
Distant metastases	

## Discussion

As primary malignancies of the great vessels are exceptionally rare, most nuclear medicine specialists and radiologists have no personal experience in diagnosing the disease. This study therefore aimed at identifying typical imaging features of primary sarcomas of the great vessels using MDCT and MRI based on our single-center experience. Furthermore, we report on advanced imaging techniques such as MR perfusion and metabolic F-18 FDG PET/CT imaging that have proven potential as tools for possible diagnosis verification and tumor staging.

To date, about 150 cases of primary aortic sarcomas and approximately 250 cases of primary sarcomas of the pulmonary arteries have been reported in the English-language world literature [1-3]. Many of these reports focus on the clinical presentation or pathology. Despite the rareness of these tumors it is important for the radiologist to bear this seldom differential diagnosis in mind, especially when alerted by typical imaging findings that raise the suspicion of a neoplastic origin of an intravascular filling defect as presented in this manuscript.

The overall prognosis of patients with sarcomas of the great vessels is poor. Many patients have a locally advanced state or distant metastases at the time of presentation [1-3]. Hence, a timely diagnosis is essential to identify the patients in a stage of disease where an aggressive surgical therapeutic approach with curative intent is still feasible. We observed a median delay in diagnosis of 11 weeks between the first imaging study and the final diagnosis. The awareness of typical imaging findings presented herein may add to a shortening of this undesirable diagnostic delay. However, the large interval between the first consultation of a physician and the final diagnosis in three of our cases is assumably of higher prognostic relevance, but can hardly be reduced due to the unspecific symptoms that mimic more common diseases such as pulmonary hypertension or congestive heart failure [1,5,7,8].

With regard to primary malignancies of the pulmonary arteries we observed a large filling defect with vascular distension, unilaterality of the filling defect and a lack of improvement despite effective anticoagulation as the major imaging findings suggestive of malignant disease. These findings are usually the first ‘red flags’ that can possibly be encountered in an MDCT scan of the chest usually acquired for unspecific clinical symptoms such as dyspnea or chest pain. Our results are well in concordance with earlier observations in the literature. Yi et al. reported comparable findings in a group of seven patients in a study on the MDCT-appearance of pulmonary artery sarcomas [7]. However, they observed extraluminal expansion in 5 of 7 patients (71%) as compared to 25% (2/8) in our cohort. This difference may be due to an advanced tumor stage in Yi’s patient group as the findings occurred in our two patients in the follow-up examinations showing progressive disease. Furthermore, Yi et al. may have had more patients with the mural form of the disease in his patient group as opposed to the exclusively luminal form in our patients. Fasse et al. have also seen comparable findings in five patients [8]. We did not observe a bilateral involvement of the central pulmonary arteries, however, it may occur as shown in a case by Simpson and Mendelson [9].

With respect to sarcomas of the aorta we identified an atypical location of a thrombus and a pedunculated shape as characteristic morphologic imaging features in two patients of our cohort, consistent with the intimal form of aortic sarcoma as described in the literature. Comparable findings have been reported by Bendel et al. [3]. Our single case of a patient with the mural disease pattern who had previously undergone aortic surgery is well in concordance with a few reports in the literature that describe the development of aortic sarcoma in patients after open or endovascular aortic repair. Whether the graft itself or a chronic perigraft infection might contribute to the induction of malignancy in the aortic wall remains subject to discussion, as the number of cases is very limited [10,11]. One patient of our cohort was diagnosed with a sarcoma of the pulmonary vein, which is even less frequently reported in the literature than pulmonary artery sarcoma [12].

When an atypical vascular filling defect is noticed base on the criteria described above, further imaging workup is usually recommended to verify the suspicion of malignant disease. Different imaging modalities such as dynamic MDCT and MRI examinations for the evaluation of lesion vascularization or metabolic imaging using F-18 FDG PET(/CT) are possible examinations whose potential benefits have been sporadically described in the literature. There is only sparse data on the role of F-18 FDG PET(/CT) for the imaging of primary sarcomas of the great vessels, mainly in patients with pulmonary artery involvement. However, the few cases reported in the literature are promising [13-16]. Ito et al. reported on three patients with pulmonary artery sarcomas and demonstrated that the mean SUV_max_ of 7.6 was significantly higher than in patients with pulmonary embolism [13]. Wittram and Scott observed SUV_max_ values in the range from 0.45 to 3.03 for acute pulmonary emboli, which is considerably lower than the SUV_max_ values reported for pulmonary sarcomas [17]. Another small series of three patients was published by Tueller et al. with an SUV_max_ of 5.2 (reported only for one patient) [16]. Treglia et al. recently reported an SUVmax of 13 for a primary pulmonary epitheloid angiosarcoma that presented as an intrapulmonary mass [18]. There is even less experience in patients with aortic sarcoma [19,20] or sarcoma of the pulmonary veins [21]. Our own results on three patients are also encouraging. We observed unequivocal positive findings in all four patients that underwent a F-18 FDG PET or F-18 FDG PET(/CT) examination. Our mean SUV_max_ of 13.1 for patients with pulmonary artery disease was even higher than the values reported in the literature. As PET/CT usually is a whole-body examination it does not only help to decide on the possibly malignant nature of a filling defect, but at the same time serves as a whole-body staging modality. In one of our cases, F-18 FDG PET(/CT) was able to demonstrate embolic metastases in the lungs as well as a peripheral metastasis to the brain (Figure 5).

Although described in most reports of primary sarcomas of the great vessels, there is only few quantitative data on the degree of enhancement or perfusion of these tumors. Kacl et al. reported on dynamic MRI in four patients with sarcomas of the pulmonary arteries and found a ‘considerable variability’ of the contrast enhancement, which was interpreted as being dependent on the degree of differentiation [22]. Howarth et al. described a single case in which an MRI first-pass perfusion sequence was used to prove the vascularization of the lesion [23]. Fasse et al. reported on MDCT and MRI findings in five patients with sarcomas of the pulmonary arteries and found only MR imaging suitable for the evaluation of lesion enhancement [8]. However, tumor vascularization cannot be demonstrated in all cases [8]. In our patient population, MR demonstrated a contrast enhancement of the lesions in 3 out of 4 MRI scans of patients with pulmonary disease and in both MRI scans available in the patients with aortic tumors. Notably, the enhancement was subtle in all our patients and required a thorough analysis of the contrast-enhanced sequences. No enhancement was visualized on standard MDCT examinations, except for the patient with the extensive mural aortic sarcoma. However, we did not evaluate the possible value of dedicated MDCT perfusion techniques. Besides the evaluation of possible lesion perfusion, MRI has an additional value in the depiction of the relationship between the tumor and its surrounding structures and may assist the preoperative planning, especially when ECG-gated cine sequences are used.

A number of important limitations of our manuscript need to be addressed. First of all and most obvious, the number of patients in our study is limited and the imaging studies available for our analyses were heterogeneous and do not follow a specific protocol. However, as already outlined above, sarcomas of the great vessels are exceedingly rare and most reports in the literature are therefore limited to descriptions of single cases or small series. To the best of our knowledge, our manuscript represents the largest study group that has been evaluated with respect to the imaging findings. Second, we did not compare our imaging findings to a contrast group such as patients with pulmonary embolic disease or severe aortic atheromatosis. However, the selection of such control groups would be highly artificial. Furthermore, the number of patients in our study is too limited to calculate statistical significances or sensitivities and specificities. In addition, we did not evaluate the potential role of transthoracic or transesophageal echocardiography in our patient cohort. Depending on the location, these modalities may also serve as a viable tool for the depiction of the tumor. However, as the field of view and the capability to characterize soft tissues are limited, echocardiography has its strongest potential in patients with cardiac sarcomas and is inferior to MDCT and MRI in cases of extracardiac disease [3].

## Conclusion

In conclusion, if an intraluminal filling defect of a great vessel that is incidentally detected on a routine MDCT or MRI scan shows the characteristic imaging findings outlined in this manuscript, primary sarcoma should be taken into consideration and further diagnostic workup is recommended to avoid any delay in the diagnosis of this rare disease. MR perfusion techniques may add information on the nature of the lesion but the findings may be subtle and equivocal. Based on our limited experience, we see a potential role of F-18 FDG PET/CT in these patients and suggest it to be considered as part of the imaging workup in these patients.

## Competing interests

The authors declare that they have no competing interests.

## Authors’ contributions

CvF, BM and CF designed the study, identified the patients to be included in the study and carried out the analysis. CvF and TR drafted the manuscript. FL carried out the histopathologic studies. FBe, FWa and TR participated in the design and coordination of the study and helped to draft the manuscript. All authors read and approved the final manuscript.

## Pre-publication history

The pre-publication history for this paper can be accessed here:

http://www.biomedcentral.com/1471-2342/13/25/prepub
